# Supplementary figures and images for: Lack of Paxillin phosphorylation promotes single-cell migration in vivo
Source: J Cell Biol. 2023 Feb 1;222(3):e202206078. doi: 10.1083/jcb.202206078 (PMC9929932; doi:10.1083/jcb.202206078)

Paxillin

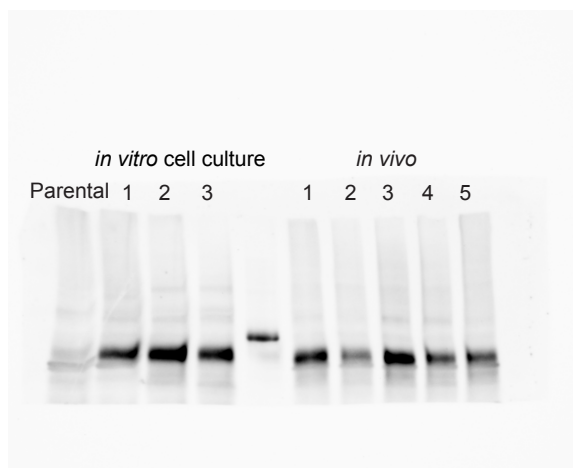

pY118  
Paxillin

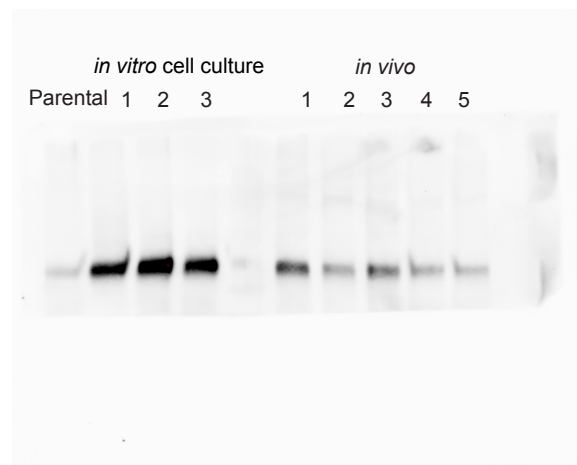

GFP

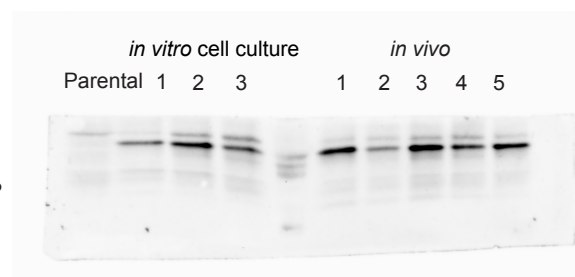

Supplement: SourceData F3 — is the source file for Fig. 3. [file JCB_202206078_SourceDataF3.pdf]

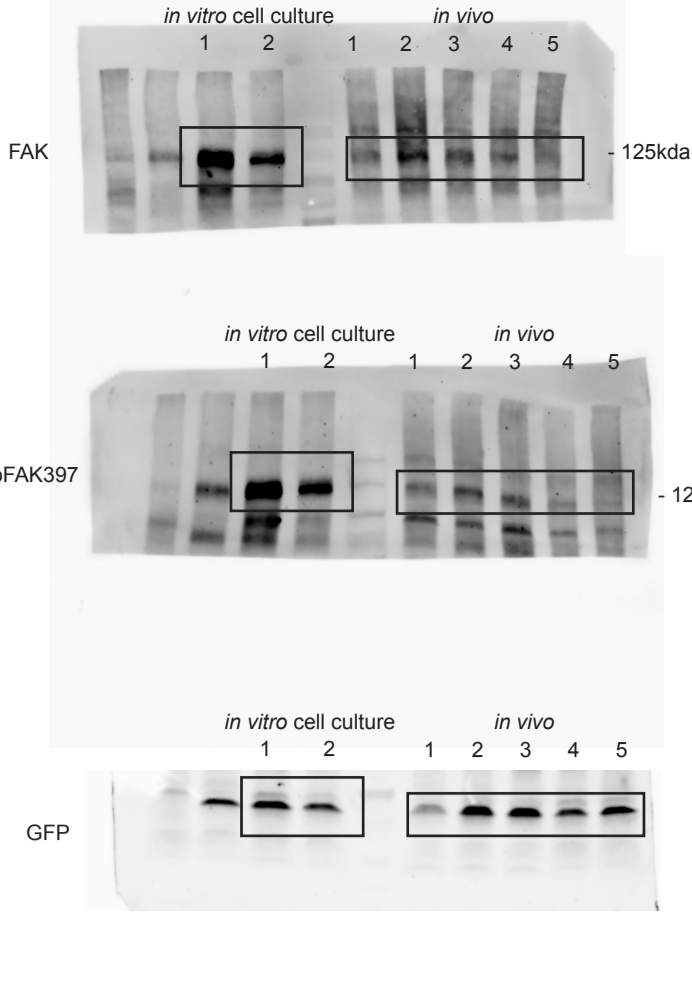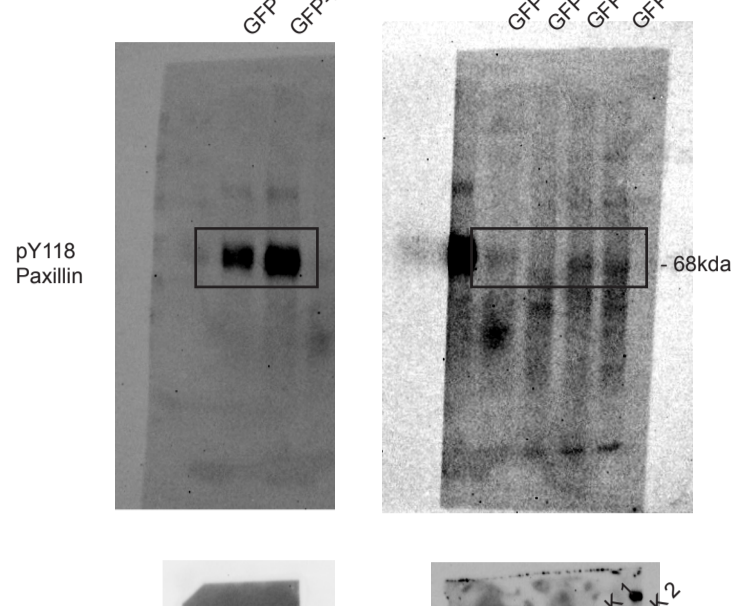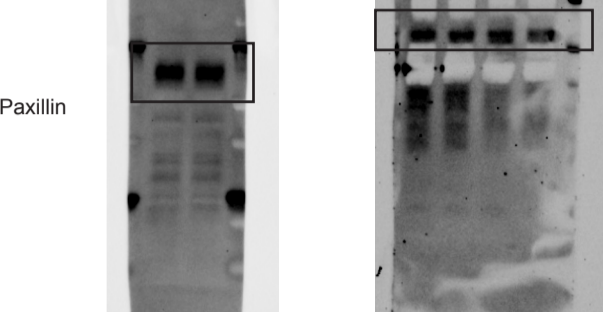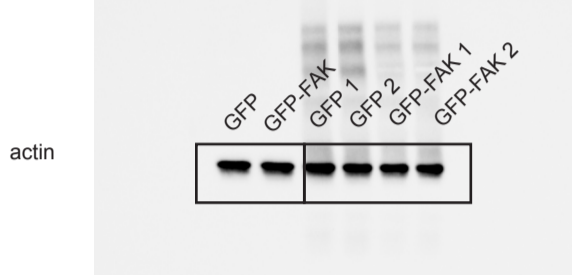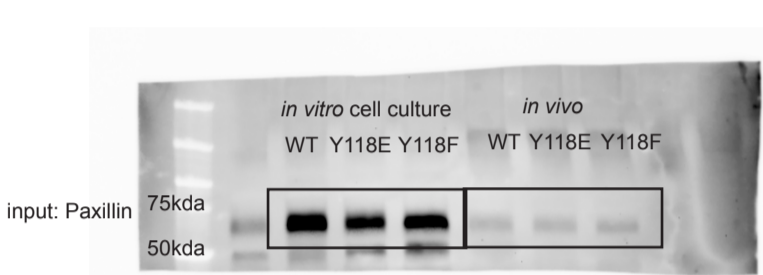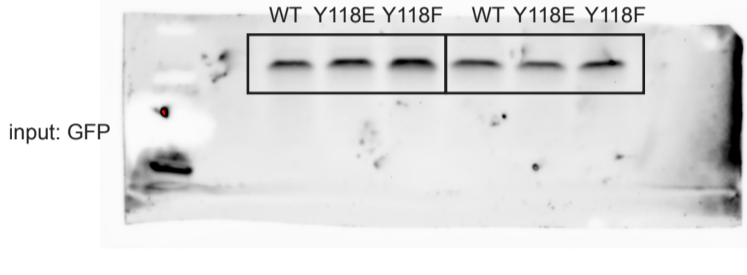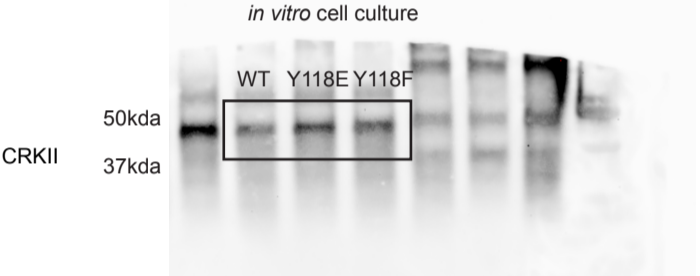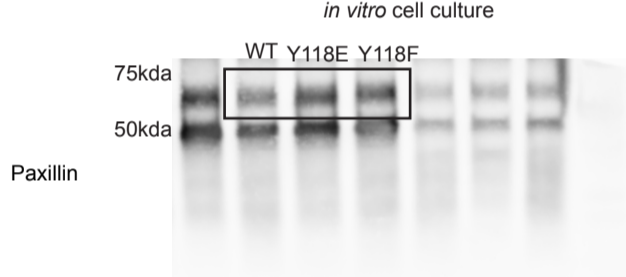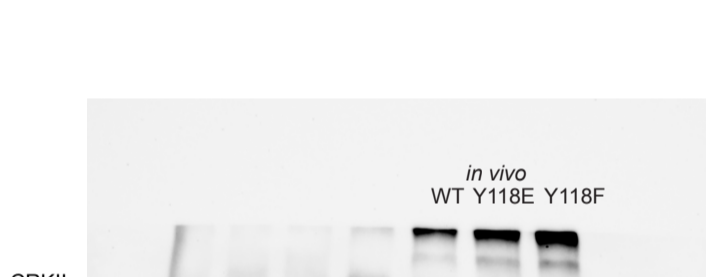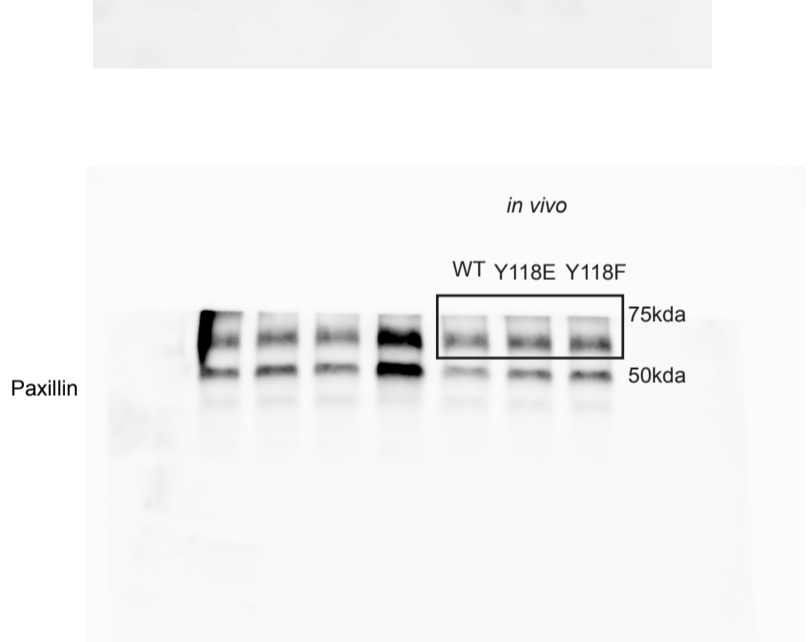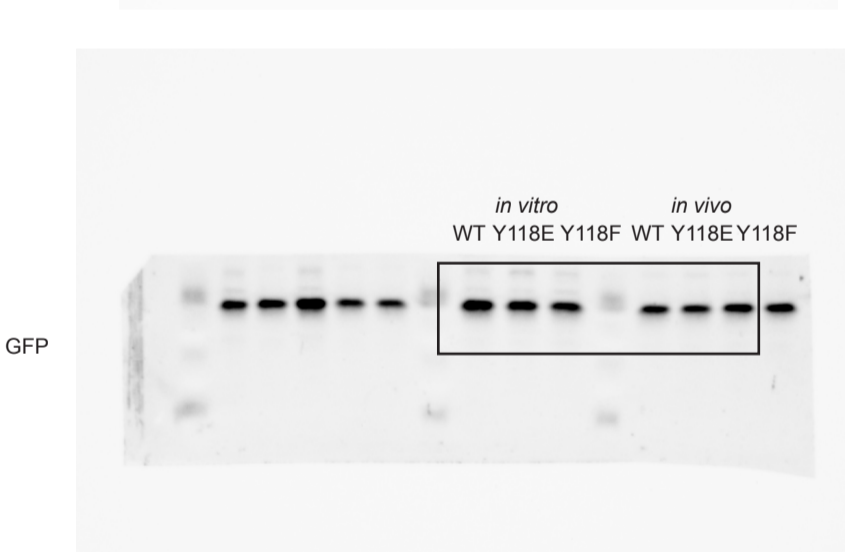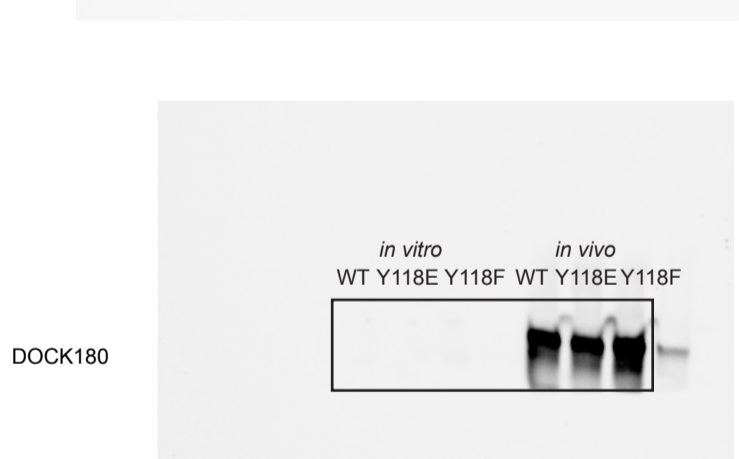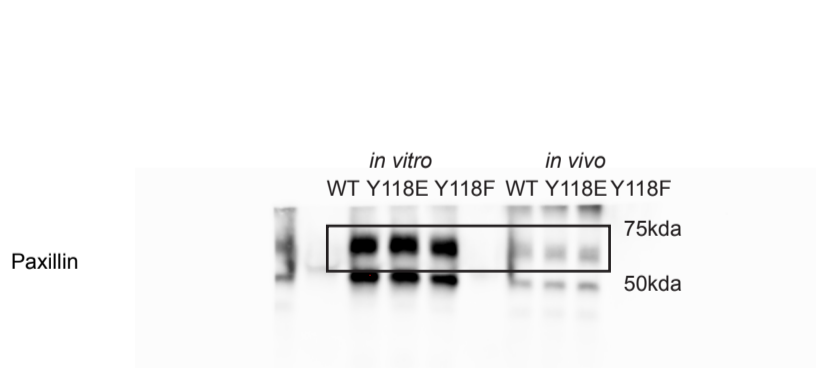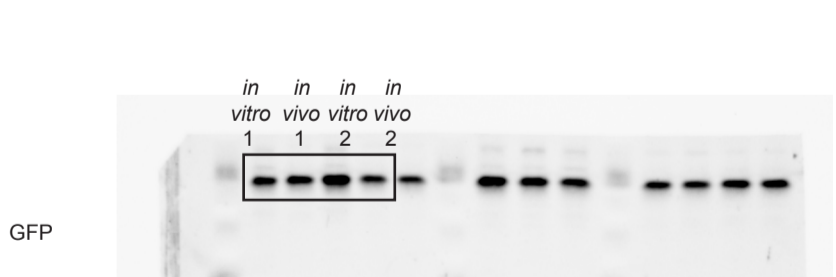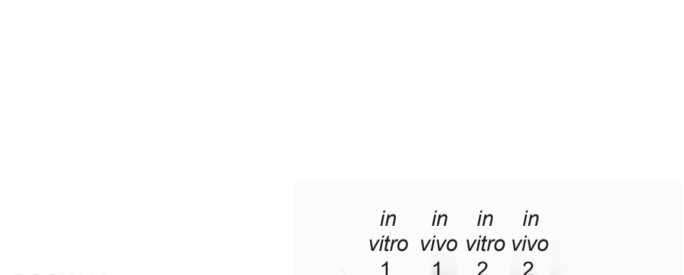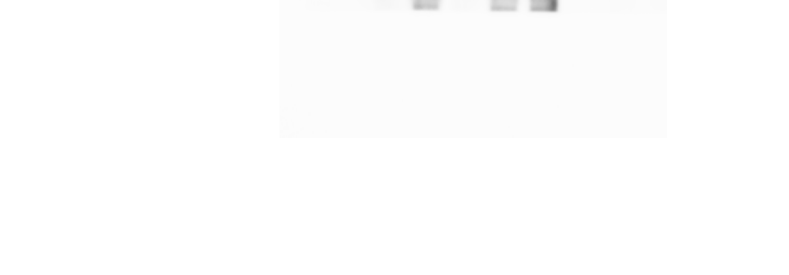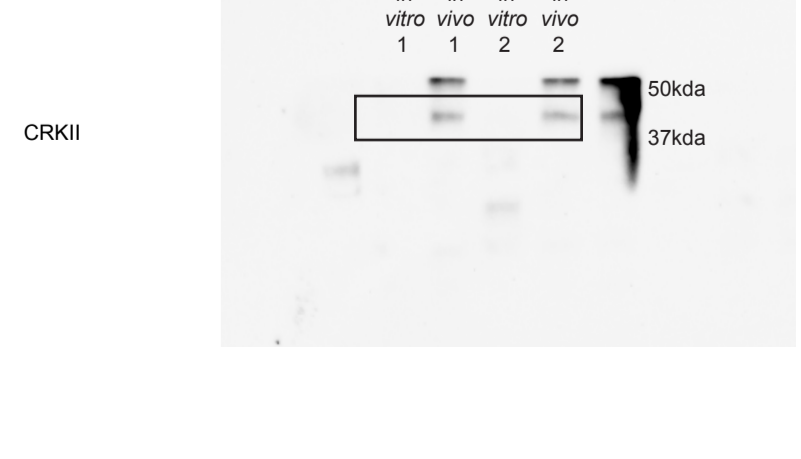

Supplement: SourceData F5 — is the source file for Fig. 5. [file JCB_202206078_SourceDataF5.pdf]

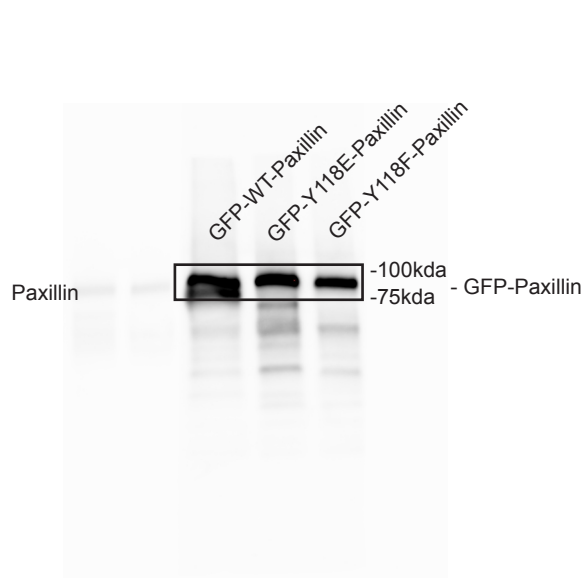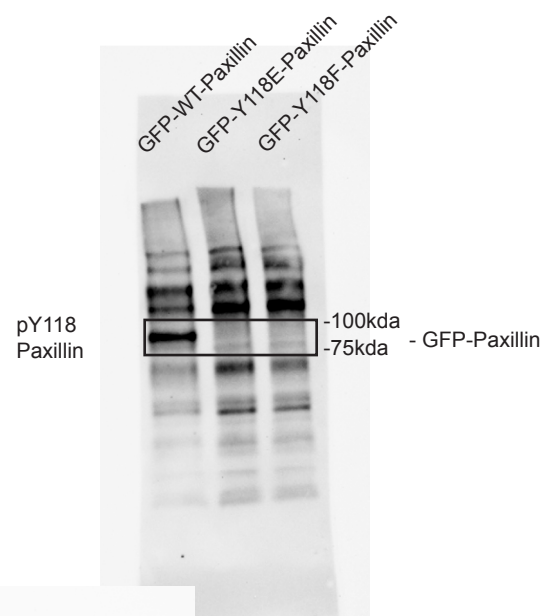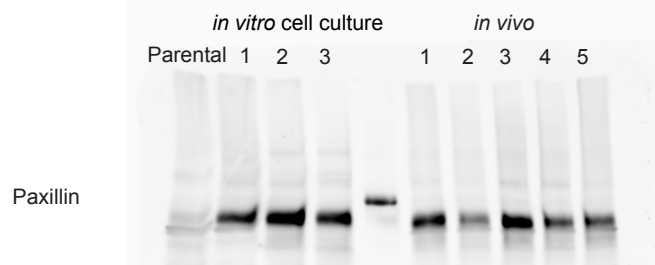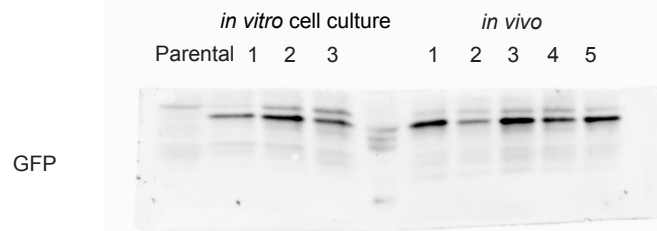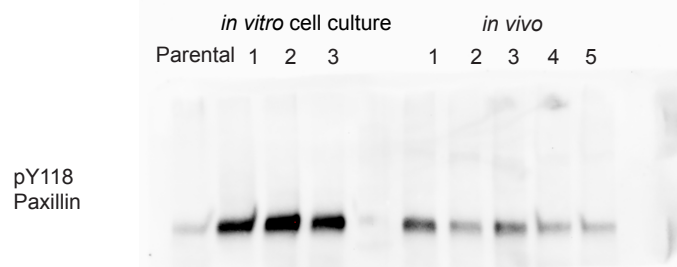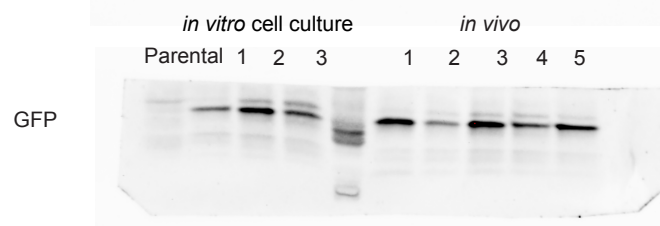

Supplement: SourceData FS2 — is the source file for Fig. S2. [file JCB_202206078_SourceDataFS2.pdf]

Paxillin

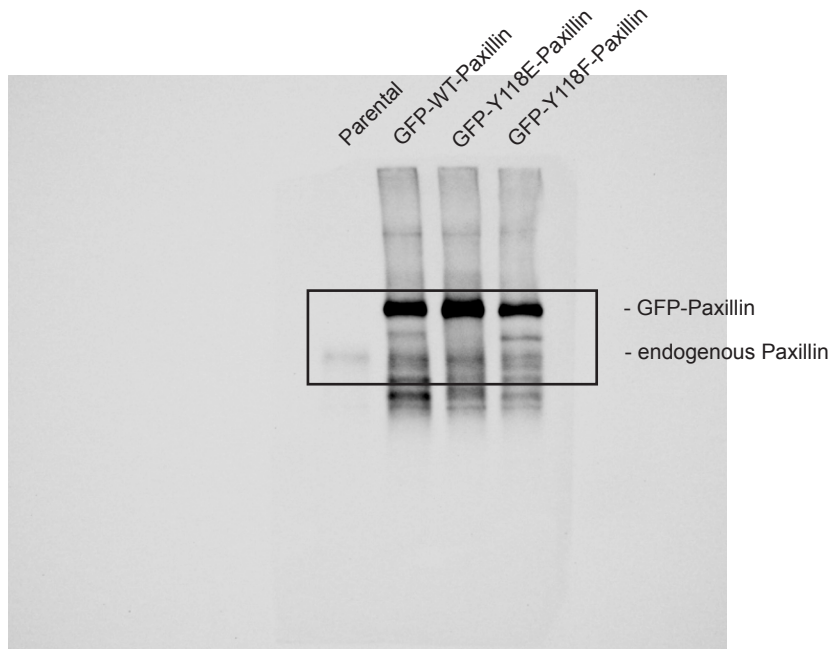

GFP

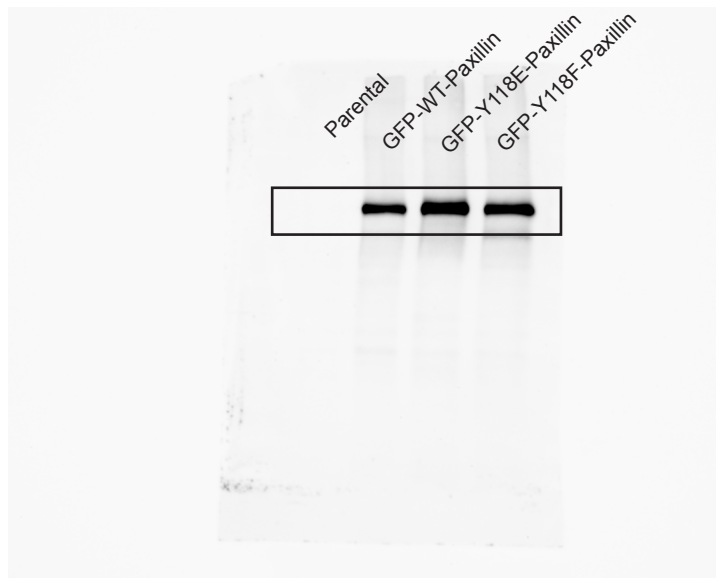

actin

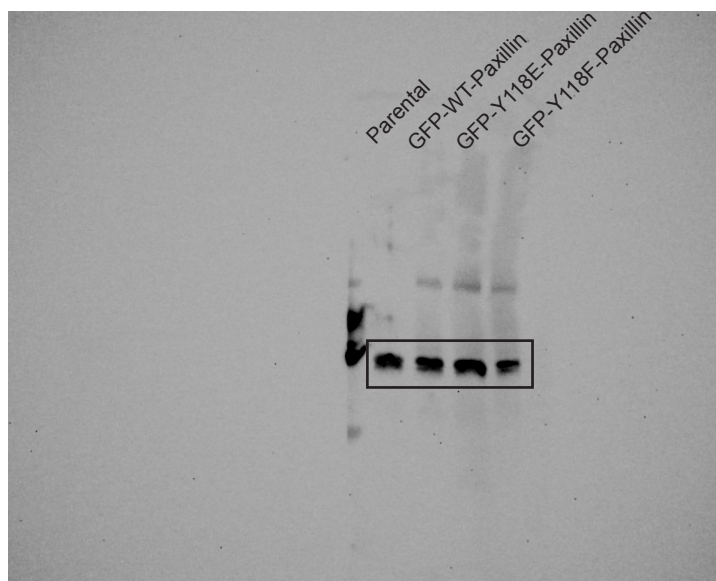

Supplement: SourceData FS3 — is the source file for Fig. S3. [file JCB_202206078_SourceDataFS3.pdf]

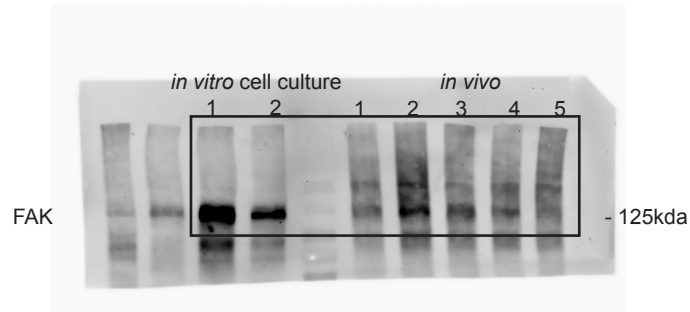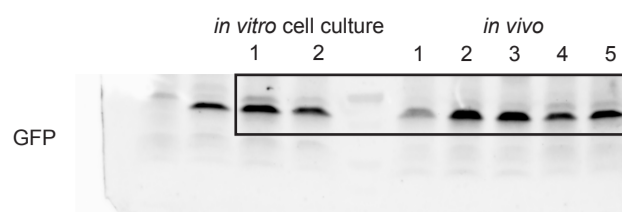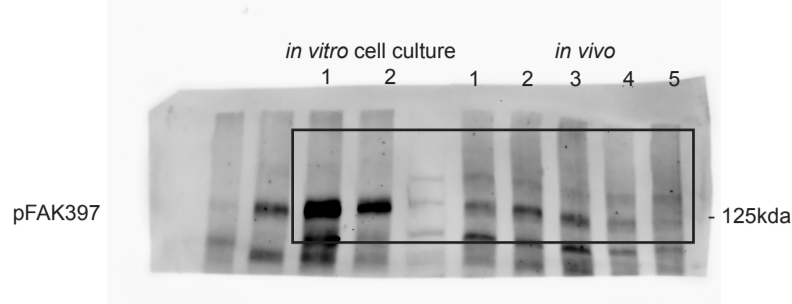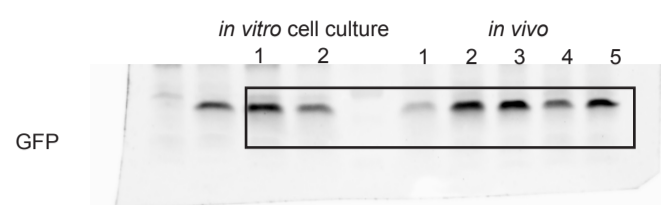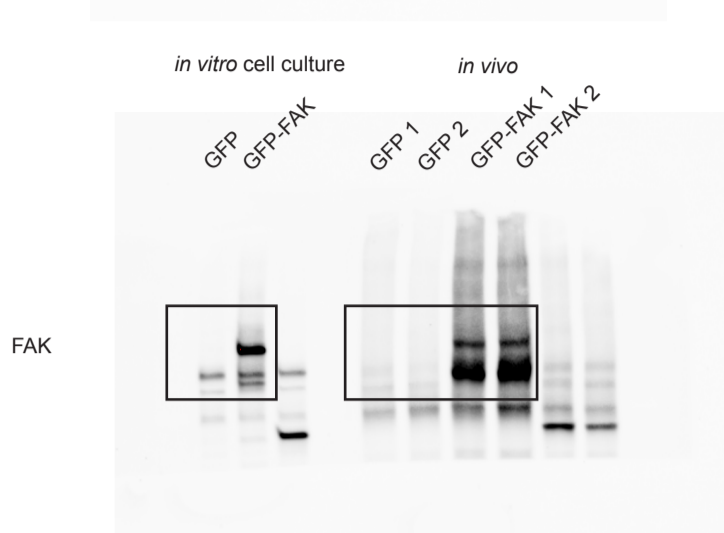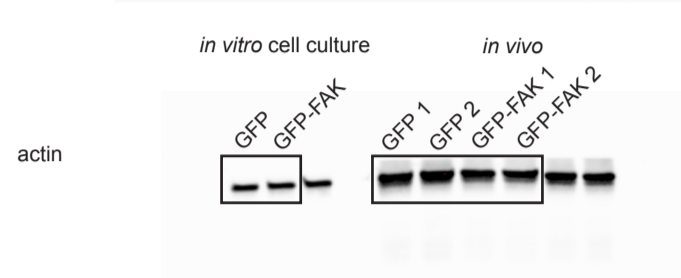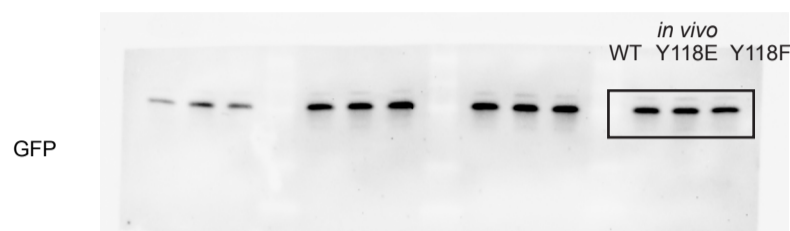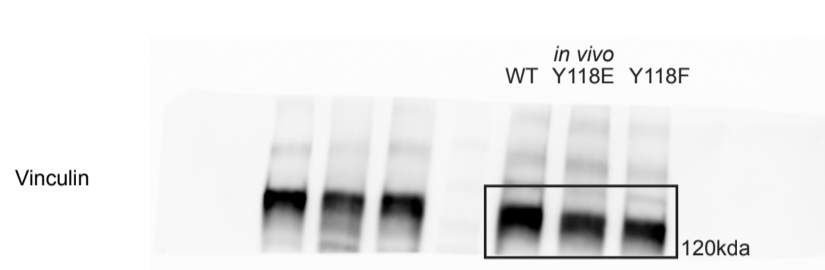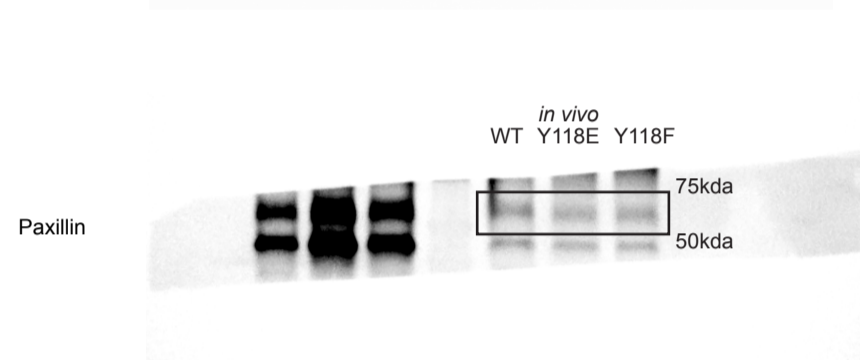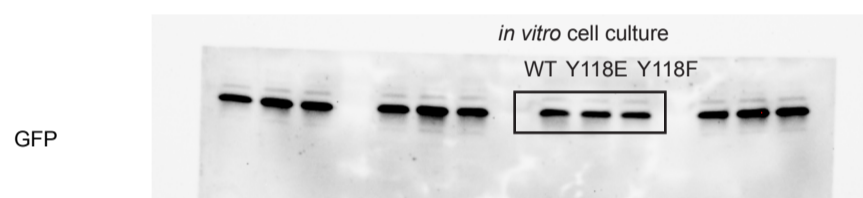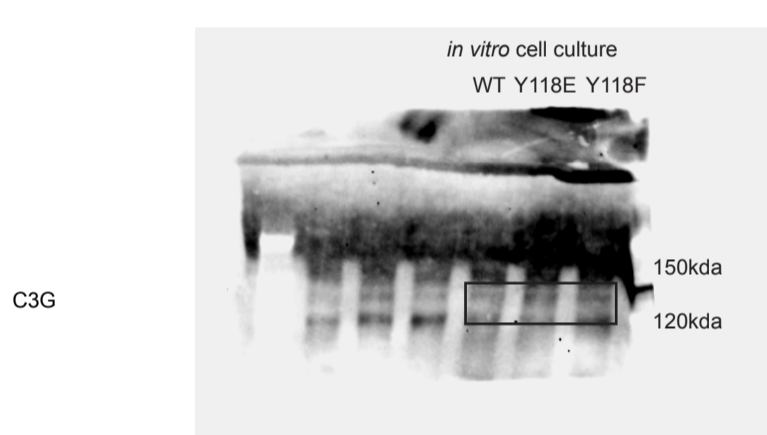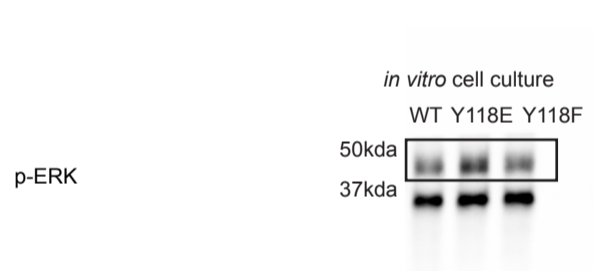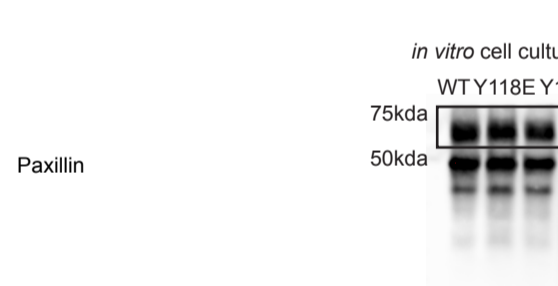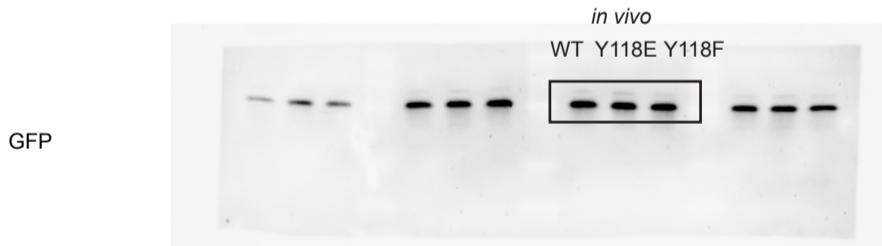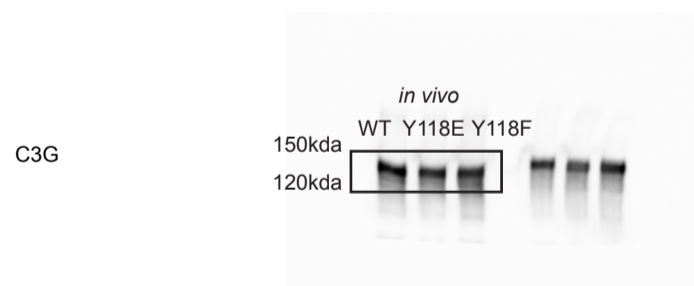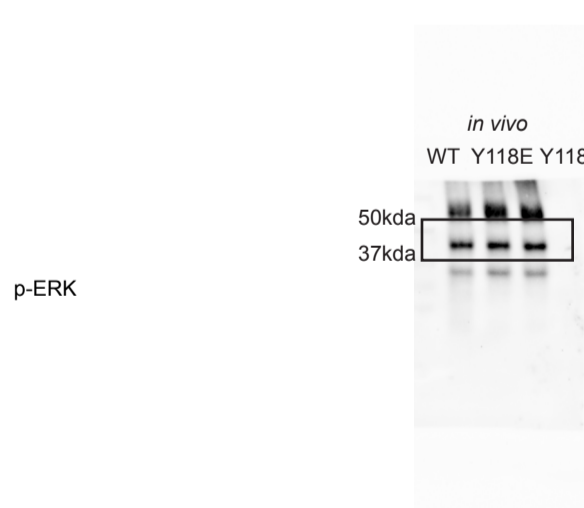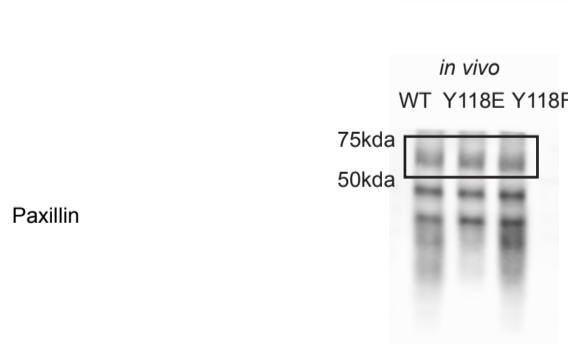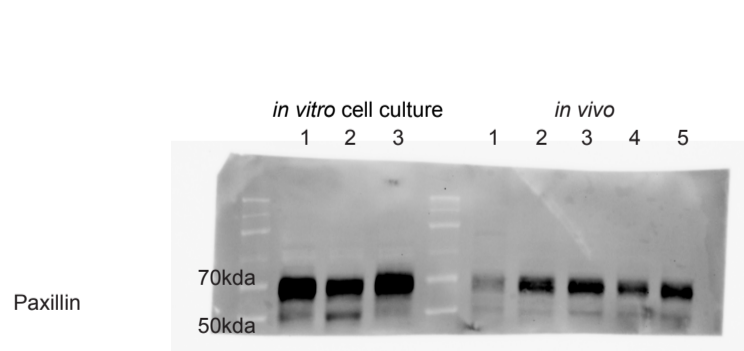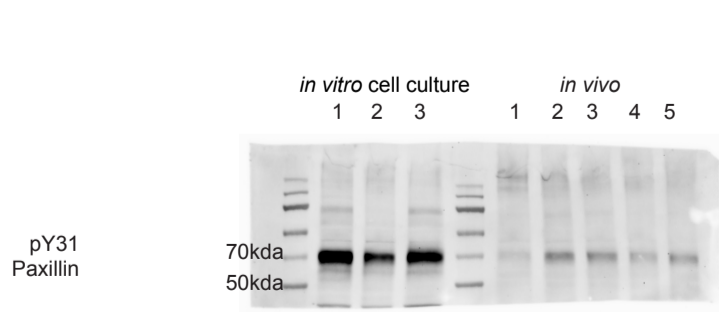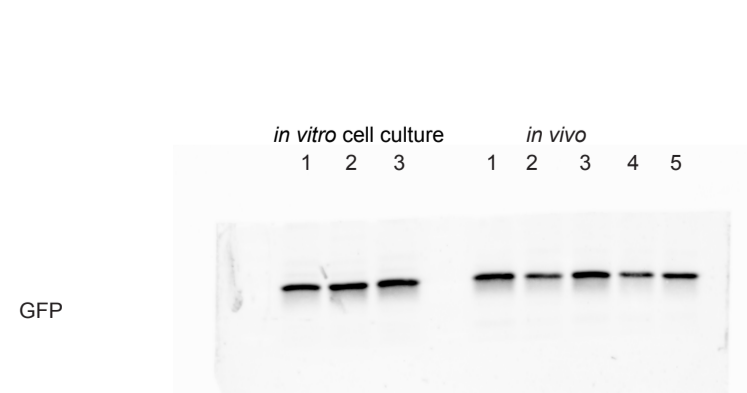

Supplement: SourceData FS5 — is the source file for Fig. S5. [file JCB_202206078_SourceDataFS5.pdf]
